# Local co-expression of GLP1R and INS in human cortical interneurons

**DOI:** 10.3389/fendo.2026.1788432

**Published:** 2026-03-27

**Authors:** Nóra Faragó, Katalin Á. Kocsis, Sándor Bordé, Ágnes Zvara, Pál Barzó, László G. Puskás, Gábor Tamás, Éva A. Csajbók

**Affiliations:** 1 HUN-REN-SZTE Research Group for Cortical Microcircuits, Department of Physiology, Anatomy and Neuroscience, University of Szeged, Szeged, Hungary; 2 Laboratory of Functional Genomics, Core Facility, HUN-REN Biological Research Centre, Szeged, Hungary; 3 Department of Neurosurgery, University of Szeged, Szeged, Hungary; 4 Anthelos Ltd., Szeged, Hungary; 5 Endocrinology and Diabetology Center, Department of Medicine, Albert Szent-Györgyi Medical School, University of Szeged, Szeged, Hungary

**Keywords:** GLP1R, INS, mRNA, single cell mRNA sequencing, human cortical interneurons, neurogliaform cell, rosehip cell

## Abstract

**Aims/hypothesis:**

Emerging evidence suggests that glucagon-like peptide-1 receptor (GLP1R) and insulin (INS), traditionally associated with peripheral metabolic regulation, also exert key functions in the central nervous system. We hypothesised that specific human cortical interneuron subtypes locally express GLP1R and INS, a molecular feature that may be relevant for exploring potential intracortical metabolic signalling mechanisms.

**Methods:**

We analysed single layer 1 GABAergic interneurons microdissected from human cortical tissue using laser capture microdissection. Transcriptomic subtype identification was performed using digital PCR preamplification of LAMP5, SV2C and PRSS12 markers. GLP1R and INS and mRNA copy numbers were quantified using single-cell digital PCR, and spatial expression patterns were validated using RNAscope Hi-Plex *in situ* hybridisation.

**Results:**

Neurogliaform (LAMP5+, SV2C+, PRSS12–) and rosehip cells (LAMP5+, SV2C+, PRSS12+) exhibited significantly higher GLP1R and INS expression than other LAMP5 interneurons. GLP1R mRNA was found in 44/72 neurogliaform and 18/36 rosehip cells, whereas INS mRNA was detected in 29/72 and 11/36 respectively. No INS expression was detected in other LAMP5 interneurons. Co-expression analysis revealed significant statistical dependency (mutual information = 0.244, p<0.0001), indicating non-random co-expression.

**Conclusions/interpretation:**

Human cortical neurogliaform and rosehip interneurons selectively co-express GLP1R and INS, conceptualising the existence of a local intracortical GLP1R- insulin–signalling loop. These findings provide a context for future investigations into cerebral glucose-regulatory processes implicated in certain neurodegenerative conditions, such as mild cognitive impairment in diabetes mellitus.

## Introduction

Insulin and its receptor pathways are increasingly recognised as important players in central nervous system (CNS) function, beyond their classical roles in peripheral glucose metabolism. Experimental evidence indicates that insulin (INS) influences synaptic plasticity, neuronal survival, and cognitive performance (1, 2). Glucagon-like peptide-1 receptor (GLP1R) activation has been firmly established as a trigger of insulin secretion in pancreatic β-cells through early studies in isolated islets (3), perfused pancreas (4), and β-cell models that defined its insulinotropic action, cAMP-dependent signalling (5), receptor binding (6), and molecular identity (7).

Insulin- and GLP1-receptors are expressed across multiple brain regions, where experimental studies implicate them in processes related to synaptic plasticity, neuronal survival, and cerebral glucose utilisation. Disruption of insulin-related signalling has been consistently reported in post-mortem brain tissue from individuals with mild cognitive impairment (MCI) and Alzheimer’s disease (AD) (8), supporting the concept that impaired INS-associated pathways represent an early feature of cognitive vulnerability (9).

In parallel, GLP1R has emerged as a potential modulator of insulin-related and metabolic processes in the brain. Preclinical studies suggest that GLP1R activation may influence neuroinflammatory responses, mitochondrial function, and insulin signalling cascades (10), while clinical investigations of GLP-1 receptor agonists (GLP-1RAs) in MCI and early AD have yielded heterogeneous outcomes, indicating that central effects are likely context- and cell-type dependent (11–13).

Characterising the expression pattern of GLP1R and INS in human cortex may help refine conceptual models of cerebral glucose-regulatory processes relevant to neurodegenerative diseases, without presupposing their functional interactions.

Despite this growing interest, the cellular and spatial localisation of GLP1R and INS within defined neuronal subtypes of the human cortex remains poorly characterised.

In our previous rodent studies we found that insulin is strongly expressed in GABAergic neurogliaform cells (NGFs) in the cerebral cortex of the rat, detected by single-cell digital PCR. Focal application of glucose or glibenclamide to NGFs mimics the excitation suppressing effect of external insulin on local microcircuits via insulin receptors (14). We have also proven the co-expression of *Glp1-receptor (Glp1r)* and *insulin (Ins2)* mRNA in rat GABAergic NGFs, with a much higher copy number in hyperglycaemia than in hypoglycaemia (9.6 times higher, p < 0.008). The functionality of the Glp1-receptors was confirmed with whole-cell patch-clamp electrophysiology, showing a reversible effect of GLP-1 on NGFs. This effect was prevented by pre-treatment with the GLP-1 receptor-specific antagonist exendin-3 (9–38) and was absent in hypoglycaemia (15).

Based on this rodent data we aimed to perform a descriptive, cell-type–resolved analysis of the local expression patterns of *GLP1R* and *INS* in human cortical interneurons (16).

GABAergic interneurons are a diverse class of cortical neurons that regulate network excitability and information processing and are commonly classified into major subtypes based on molecular markers such as parvalbumin (PVALB), somatostatin (SST) and vasoactive intestinal peptide (VIP). The identity of cortical interneuron subtypes is broadly conserved between rodents and primates including humans, with crucial quantitative and qualitative differences. Interestingly, a severalfold increase in the ratio of inhibitory interneurons from mouse to human was detected by Loomba et al. (17) and relative to the 4:1 ratio of excitatory to inhibitory (E/I) neurons in rodents, the surprising 2:1 E/I neuron ratio in humans was confirmed by spatial transcriptomic studies (18–20), except in the primary visual cortex, where the cellular E/I ratio is similar to mouse (21). Further complexity to the human cortical inhibitory landscape is the recent identification of cell types specific to human (22). Rosehip cells (RHC) emerged as a novel, human-specialised GABAergic interneuron subtype located in layer 1. RHCs are distinguished by their compact arborisation, dense axonal boutons resembling rosehips, and unique transcriptomic profiles, including expression of lysosomal associated membrane protein family member 5 (LAMP5), synaptic vesicle glycoprotein 2C (SV2C), and protease serine 12 (PRSS12) (22). NGFs, while more conserved across species, also robustly express LAMP5 alongside SV2C but lack PRSS12, making these markers useful for molecular discrimination between the two subtypes (23). Notably, LAMP5-positive interneurons comprise approximately 20% of layer 1 inhibitory neurons in the human neocortex, compared to just 5–10% in the mouse, highlighting a significant species difference in this cell population (23, 24). RHCs are thought to regulate apical dendritic input to pyramidal neurons and have no known analogues in rodent models. Their exclusive presence and elevated abundance in humans underscore the need to investigate human-specific neuronal mechanisms, particularly in the context of metabolic signalling pathways that may affect cognition and plasticity (25).

In this study, we used laser capture microdissection and digital PCR to profile single interneurons from layer 1 of the human cerebral cortex. We focused on NGFs and RHCs - two molecularly distinct subtypes of interneurons - investigating the expression of GLP1R and INS mRNA and their co-localisation.

Our findings provide a cell-type–specific and spatial description of INS GLP1R and INS mRNA expression in human cortical interneurons, offering an anatomical framework for future studies investigating potential roles of metabolic signalling in cortical circuits.

## Methods

### Cryosectioning and staining

Small pieces of human brain cortex of about 0.1-0.5 cm were cut from patients, embedded in Tissue-Tek O.C.T. Compound, immediately frozen in liquid nitrogen and stored at -80 °C until sectioning. Brain cortex sections of 10 μm thickness were sectioned with a Leica cryostat under a -20 °C setting, transferred individually to Super Frost Plus slides, dried inside the cryostat for 1-2min, and stored at -80 °C. The frozen sections were immersed immediately in 75% ethanol for 30 seconds. After fixation, the slides were stained in 1% warmed cresyl violet solution (Sigma-Aldrich, Taipei City, Taiwan) for 60 seconds. Then, the sections were dehydrated in ascending series of ethanol, treated with xylene for 5 minutes. Importantly, all steps, except the incubation in xylene, were performed using ice-cold reagents. The time to dissect neurons of one dehydrated cryosection should not be longer than 20–30 min to avoid rehydration of the tissue sections which would result in RNA degradation.

### Laser capture microdissection

Cells in the first layer were microdissected using a PALM MicroBeam instrument (Zeiss, Oberkochen, Germany) employing the CloseCut Auto Laser Pressure Catapulting mode. The microdissection process was visualised with an AxioCam ICC camera coupled to a computer and was controlled by Palm RoboSoftware (Zeiss, Oberkochen, Germany). Isolated cells were pressure-catapulted into PCR tube caps containing 2.5 µl 1X Single Cell Protect TM (Avidin Ltd., Szeged, Hungary) buffer facing down ward for storage. To avoid dripping and evaporation of the buffer, PCR tubes were kept at −20 °C and catapulting was performed when the buffer transitions from frozen to liquidstate (between 10 and 20 s after removal from the fridge). After collection, the tubes were closed and immediately stored at −80 °C. The completion single neuron dissection from four cryosections required no longer than 30–40 min.

### Single-cell reverse transcription and preamplification of human cortical neurons

Reverse transcription of individual microdissected cells was carried out in two steps. The first step was performed for 5 min at 65 °C in a total reaction volume of 5 μl containing the cell captured in 2.5 μl1X Single Cell Protect TM (Avidin Ltd), 0.3 μl TaqMan Assays (Thermo Fisher), 0.3 μl 10 mM dNTPs (Thermo Fisher), 1 μl 5× first-strand buffer, 0.3 μl 0.1 mol/l DTT, 0.1 μl RNase inhibitor (Thermo Fisher), and 100 U of Superscript III reverse transcriptase (Thermo Fisher). The second step of the reaction was carried out at 55 °C for 1 h, and then the reaction was stopped by heating at 75 °C for 15 min. The reverse transcription reaction mix was stored at −20 °C until preamplification. Analysis of many low abundant neuronal marker genes in the case of single cells requires multiplexing or preamplification to determine different cell types. Digital PCR reactions were carried out after preamplification of 20% of cDNA in a total volume of 10 μl (1 µl RT product, 0.5 µl of Taqman^®^ primer mix (lamp5 sv2c, prss12, rps18)5 µl TaqMan^®^ PreAmp Master Mix (Life Technologies) and 3.5 µl nuclease-free water) in MyGenie 32 ThermalBlock (Bioneer Corporation, Daejeon, South Korea) with the following cyclying protocol: 10 min hot start at 95 °C, 2 min at 55 °C, 2 min at 72 °C followed by 13 cycles (15 sec at 95 °C, 4 min at 60 °C), followed by 10 mins at 99 °C inactivation.

After preamplification QRT-PCR was carried out using LightCycler^®^ Nano Real-Time PCR Instrument (Roche) in a total volume of 20 µl containing 1 µl preamplificated cDNA, 1 µl Taqman Assays and 10 µl qPCR BIOProbe Mix Lo-ROX (PcrBiosystems, London, UK). The following cycling protocol was used: 3 min at 95 °C followed by 50cycles (40 sec at 95 °C, 40 sec at 56 °C, and 1 min at 72 °C). The LightCycler^®^ NanoAnalysis Software (Roche) determined a cycle threshold (CT) value, which identified the first cycle at which the fluorescence was detected above the baseline. The combination of three marker genes were used to define the transcriptomical cell type. The major neuronal cell types (NGFs, rosehip cells, other interneuronal cells) in layer 1 have different gene expression patterns for known markers: NGFs express LAMP5 and SV2C (but not PRSS12). RHCs express LAMP5, SV2C and PRSS12 as well. The third cluster contains all other collected first layer interneurons with any gene expression pattern different from the previous two.

### Digital PCR

For digital PCR 80% of the cell cDNA was used to determine the copy numbers of the genes of interest. The preamplified mixture (4 μl) was divided into two parts: 2 μl was used for amplification INS and 2 μl was used for amplifying GLP1R. Template cDNA was supplemented with nuclease-free water to a final volume of 8 μl. 2 μl TaqMan Assays (Thermo Fisher), 10 μl OpenArray Digital PCR Master Mix (Thermo Fisher) were mixed to obtain a total volume of 20 μl, and the mixture was evenly distributed on four subarrays (256 nanocapillary holes) of an OpenArray plate by using the OpenArray autoloader. Processing of the OpenArray slide, cycling in the OpenArray NT cycler, and data analysis were done as previously described (26). In our dPCR amplification protocol, reactions having CT values less than 23 or greater than 33 were considered primer dimer or background signals, respectively, and excluded from the dataset.

### RNAscope Hi-Plex ISH

To follow spatial expression pattern, we applied the RNAscope Hi-Plex assay of Advanced Cell Diagnostics (ACD, Newark, CA, USA) with the following custom human probes: GLP1R (NM_002062.3, 519821-T5), INS (NM_000207.2, 313571-T7), LAMP5 (NM_012261.3, 487691-T8). All the experimental steps were carried out as described in the manufacturer’s instruction (ACD). Briefly, 10um fresh frozen human brain sections were fixed in freshly made 4% PFA for an hour at RT followed by a dehydration process in 50%, 70% and 100% ethanol and then were digested with protease IV for 30 minutes at room temperature. During the probe hybridisation process, the slide was incubated at 40 °C for 2 hours in HybEZ II Oven (ACD) under humid condition with a mixture of the 3 probes. The negative control slide (consecutive section) was stained with RNAscope Hi-Plex 12 negative control probe that is provided in the kit. After washing steps, the specific signals were amplified using RNAscope Hi-Plex Amp 1–3 reagents. Following washing cycles with provided washing buffer, slides were stained with RNAscope Hi-Plex Fluor T5–T8 reagent and were counterstained and mounted by ProLong Gold Antifade Mountant with DAPI (Thermo Fisher). For the high-resolution image, signals were captured by Leica confocal LSM using 63x objective with oil. We used 4 filters: DAPI for nuclei, FITC for GLP1R, Cy5 for INS and Cy7 for LAMP5. The images were further processed by RNAscope Hi-Plex Image Registration Software (ACD).

### Statistics

Data were presented as mean ± SD if not stated otherwise. Comparisons between multiple groups were performed using the Kruskal-Wallis test, followed by a pairwise Wilcoxon rank-sum test with Holm-Bonferroni adjustment for *post-hoc* analyses. Associations between the expression of the *GLP1R* and *INS* in different cell types were evaluated using Pearson’s correlation coefficients. We quantified the colocalisation between the GLP1R and INS by calculating the Mutual Information (MI). Before the colocalisation analysis, we mapped each cell’s gene copy number to a binary variable by replacing the values greater than zero with one. To test the significance of Mutual Information and the difference between conditional- and prior probability, we used a random shuffle test with 1000 iterations. In each iteration, we fixed one variable and randomly shuffled the other over our cell dataset. We calculated the value (MI or conditional probability) using the shuffled dataset and compared it to its unshuffled version. The p-value is the ratio of the iterations with a random value higher than the original. P-value <0.05 was considered statistically significant. Statistical analyses were conducted in R (version 4.1.2).

## Results

**Figure 1:**
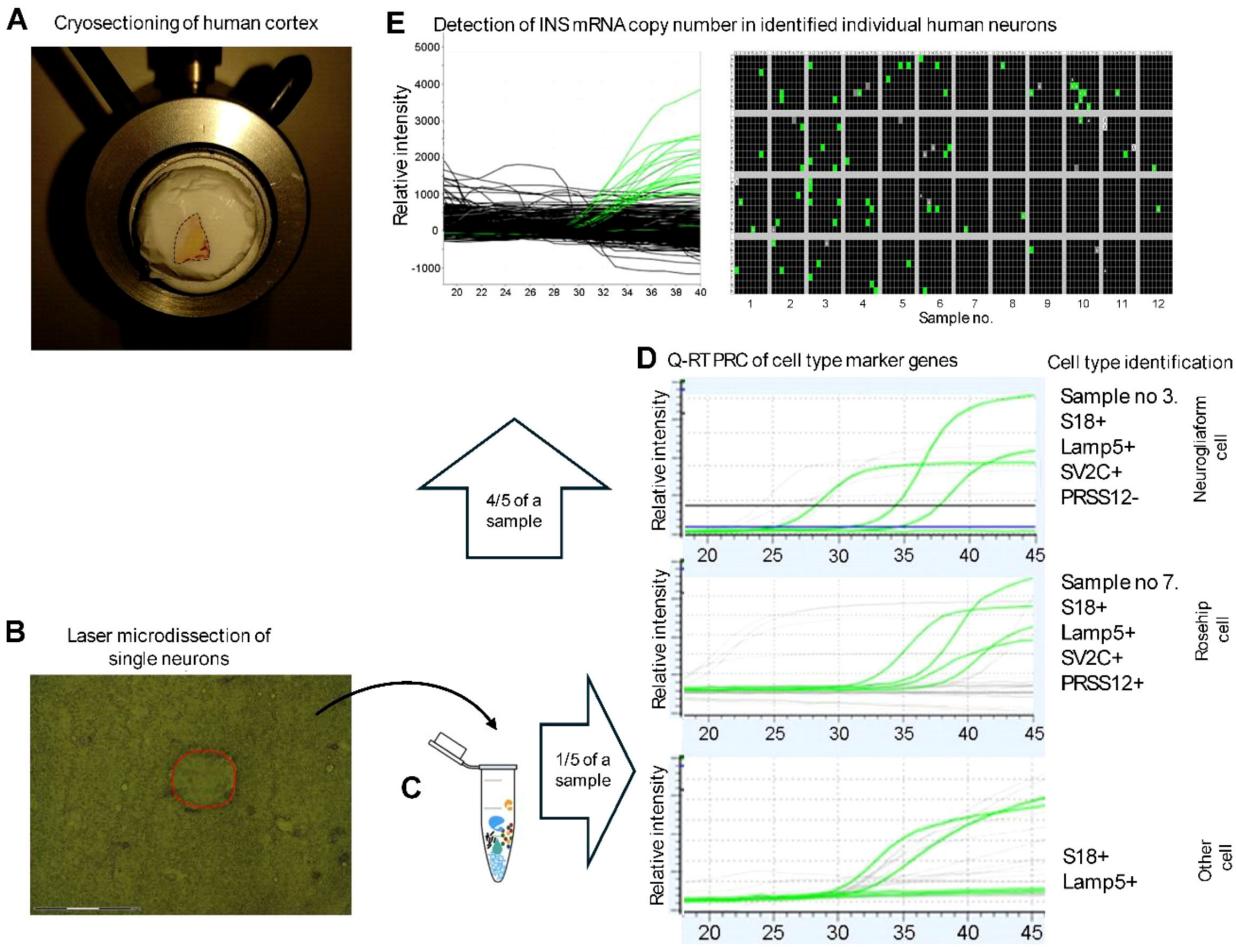
Workflow for quantitative comparison of mRNA copy numbers in identified human neuron types. **(A)**, Surgically resected blocks of human cortex are cryosectioned at a thickness remaining below the diameter of neuronal somata (10 µm). **(B, C)**, Individual neurons were laser microdissected and single cells were transferred to a fixed volume mastermix. **(D)**, One-fifth of the mastermix was used for cell-type identification by qRT–PCR analysis of marker genes. The experiment illustrates the differential identification of human neurogliaform and rosehip interneurons based on the presence or absence of LAMP5, SV2C, and PRSS12, with S18 serving as a housekeeping control. **(E)**, The remaining four-fifths of the mastermix was used for absolute quantification of mRNA copy numbers using single-cell digital PCR. *INS* mRNA copy numbers were measured across 12 single-cell samples from the same patient. Representative examples include sample 3 with 13 INS mRNA copies and sample 7 with 1 *INS* mRNA copy.

Using our previously established laser capture microdissection (LCM) protocol (27), we isolated single layer 1 interneurons from frozen cortical tissue acutely obtained during neurosurgical interventions from four neurosurgical patients without known diabetes. Clinical characteristics varied in terms of age, sex, intraoperative blood glucose levels, and diagnoses (**Supplementary Table 1**). Collected tissue was used for a pipeline of molecular analyses including transcriptomic subtype identification, RNAscope *in situ* hybridisation, and digital PCR (**Figure 1**).

**Figure 2:**
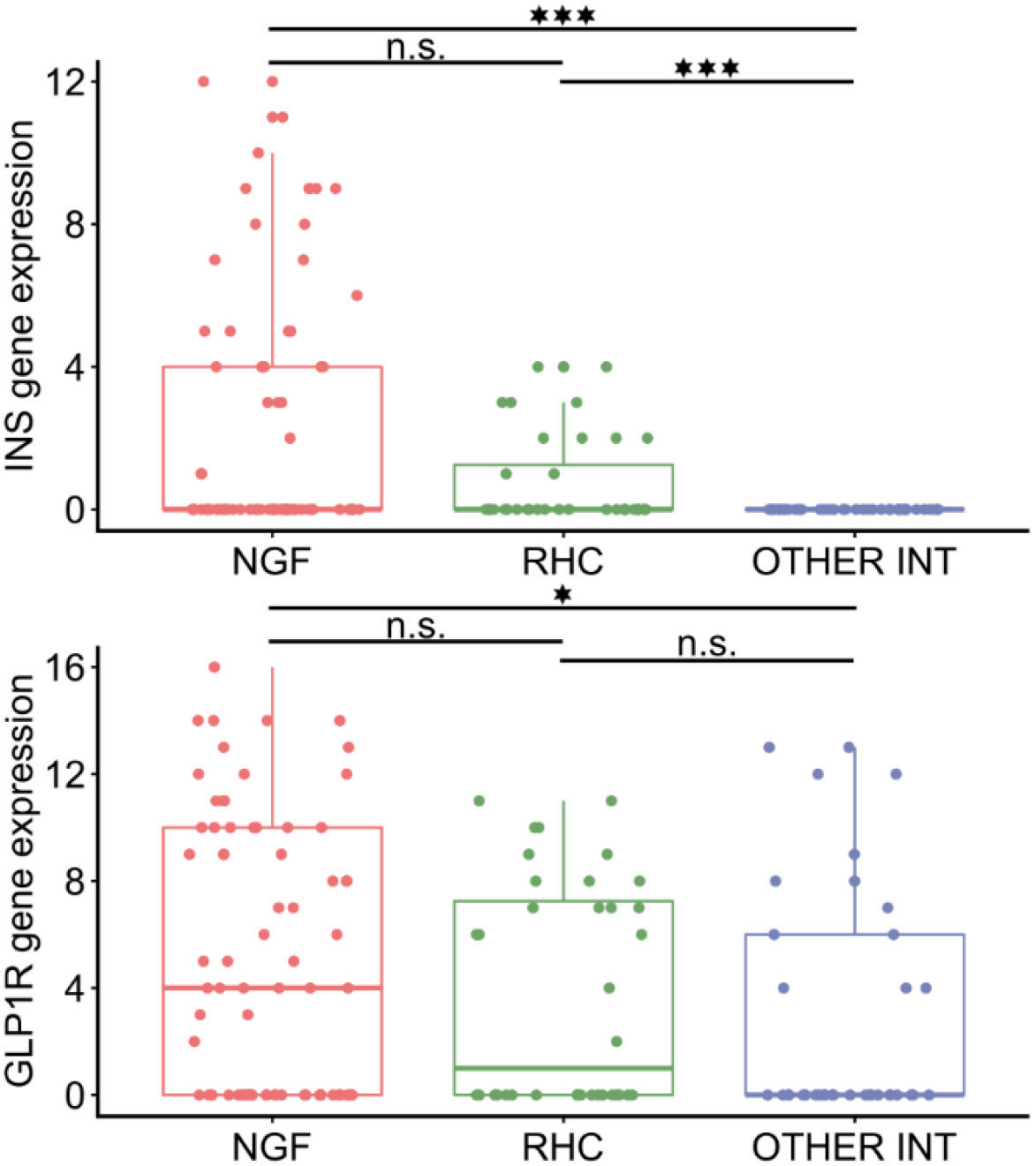
Digital PCR analysis of identified human neurons. Detection of INS (top) and GLP1R (bottom) mRNA in human neurogliaform (NGFs) and rosehip cells (RHCs). INS expression was significantly higher in NGFs and RHCs compared to other interneurons (p<0.001, Wilcoxon’s rank-sum test, Holm-Bonferroni correction), and NGF tended to be higher compared to RHC (p=0.069, Wilcoxon’s rank-sum test, Holm-Bonferroni correction). GLP1R expression was higher in NGFs compared to other interneurons (p=0.05, Wilcoxon’s rank-sum test, Holm-Bonferroni correction), but was not significantly different between NGF-RHC-other interneurons (p=0.26 and p=0.31 respectively, Wilcoxon’s rank-sum test, Holm-Bonferroni correction) ▪significant, n.s. not significant. *p=0.050, ***p<0.001.

To molecularly classify the interneurons, we developed an optimised preamplification protocol using marker gene expression. NGFs were identified by expression of *LAMP5* and *SV2C* (but not *PRSS12*), RHCs expressed *LAMP5*, *SV2C*, and *PRSS12*, while a third, heterogeneous group includes all interneurons that are LAMP5-positive but do not meet the criteria for classification as either neurogliaform or rosehip interneurons. Accordingly, this group represents a heterogeneous population of LAMP5-expressing interneurons rather than a defined subtype (23, 24). We tested the identity of cells until 12 NGFs, 6 RHCs, and 6 other *LAMP5* cells from each patient could be included in the dataset. Following cell classification, digital PCR was used to quantify low-copy-number transcripts (*INS*, *GLP1R*) in single cells across these subtypes (**Figure 2**).

*INS* mRNA was detected in 29 of 72 NGFs with a mean copy number of 2.38 ± 3.47. In RHCs, *INS* was detected in 11 of 36 cells with a mean copy number of 0.75 ± 1.27. No *INS* expression was observed in any of the 36 other interneurons. *INS* expression was significantly higher in NGFs compared to other interneurons (p<0.001) and trended higher compared to RHCs (p=0.069). Moreover, *INS* expression was significantly higher in RHCs compared to other interneurons (p<0.001).

*GLP1R* mRNA was detected in 44 of 72 NGFs with a mean copy number of 8.65 ± 2.91. It was also detected in 18 of 36 RHCs (mean copy number: 6.55 ± 2.27) and in 11 of 36 other interneurons (mean copy number: 8.15 ± 2.73). There was no significant difference in *GLP1R* expression between NGFs and RHCs (p=0.260), but *GLP1R* was more frequently expressed in NGFs compared to other interneurons (p=0.050). These results confirm a selective enrichment of *INS* expression in subgroups of LAMP5 cells and a broader but still subtype-biased expression of *GLP1R*.

We also assessed potential co-expression of *GLP1R* and *INS* mRNA. This was quantified using mutual information (MI) telling how much information two variables share, in particular whether knowing *INS* expression in a cell reduces uncertainty about *GLP1R* expression, and vice versa. This yielded an MI score of 0.244 (p<0.0001), supporting statistical dependency between *INS* and *GLP1R* expression. We also performed conditional probability analysis, testing the likelihood of one event after knowing another occurred. Our conditional probability analysis confirmed this: P(*INS*|*GLP1R*) = 0.597 vs P(*INS*) = 0.370; P(*GLP1R*|*INS*) = 0.925 vs P(*GLP1R*) = 0.574 (**Figure 2**), indicating that co-expression is not due to chance between *INS* and *GLP1R*, with a high conditional probability of one gene being expressed if the other is.

**Figure 3:**
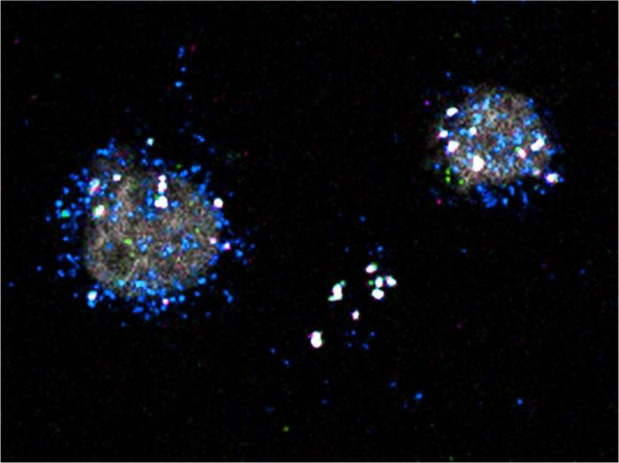
Representative image of LAMP5 (blue), INS (magenta) and GLP1R (green) expression and co-localisation in different cell subtypes.

RNAscope Hi-Plex *in situ* hybridisation experiments provided an independent spatial validation of INS and GLP1R expression within LAMP5-positive inhibitory interneurons. LAMP5 was selected as a broad marker of this interneuron class to identify the relevant population in human cortical tissue, while INS and GLP1R probes were included to confirm their local expression using an orthogonal method. We did not include additional subtype-specific markers such as SV2C or PRSS12 because the aim of these experiments was not to further refine interneuron subtypes, but to validate the presence of INS and GLP1R transcripts in LAMP5-positive interneurons more generally (**Figure 3**).

**Figure 4:**
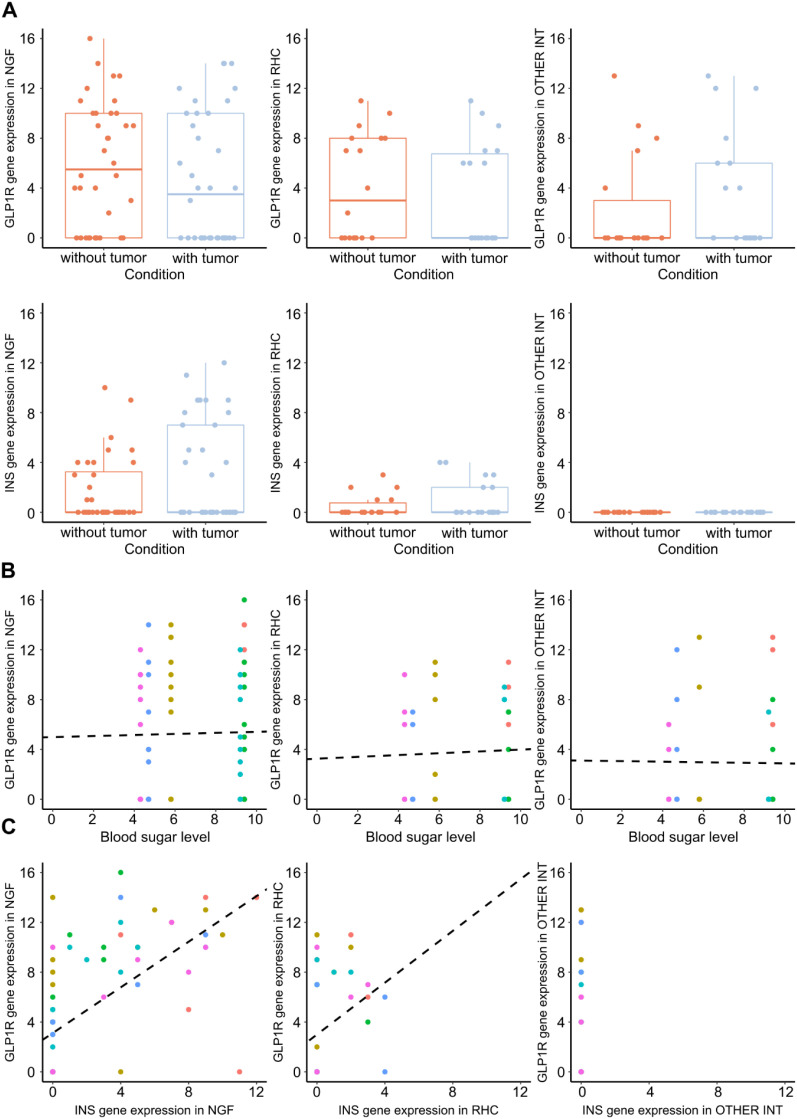
**(A)**, Comparison of *INS (bottom)* and *GLP1R* (top) mRNA expression in cells collected from patients with brain tumours to those with hydrocephalus or aneurysm (without tumour). **(B)**, *GLP1R* mRNA expression in neurogliaform (NGF), rosehip (RHC) and other cells according to blood glucose levels. No significant differences in *INS* or *GLP1R* copy numbers were found across cell types based on actual glycaemia. **(C)** Strong correlation between *INS* and *GLP1R* copy number in NGFs (p<0.001), and a trend in RHCs (p=0.059) was revealed by Pearson correlation analysis.

To assess the potential influence of pathology, we compared cells collected from patients with brain tumours (n=3) to those with hydrocephalus or aneurysm (n=3). No *INS* expression was observed in the *LAMP5+*, *SV2C-*, *PRSS12-* interneuron group in any of the patients (**Figure 4A**). In NGFs, *INS* was detected in 15 and 14 of 36 cells in tumour and non-tumour patient groups, respectively. The mean *INS* mRNA copy number in tumour patients was 7.3 ± 2.66 compared to 4.36 ± 2.62 in the non-tumour group (p=0.069). Although *GLP1R* expression in NGFs did not reach statistical significance between tumour (mean: 8.84 ± 3.69) and non-tumour patients (mean: 8.5 ± 2.78), mean copy numbers were numerically higher in tumour samples (p = 0.069). In RHCs, *INS* expression had a mean of 3.0 ± 1.87 copies per cell in tumour patients and 1.8 ± 1.43 in non-tumour patients. *GLP1R* expression averaged 6.6 ± 2.13 and 6.5 ± 2.33, respectively. No statistically significant differences were observed between the patient groups.

Patients were also stratified by intraoperative blood glucose levels: >9 mM/l (n=5) vs normal blood glucose levels (n=3).

No significant differences in *INS* or *GLP1R* copy numbers were found across cell types based on actual glycaemia (**Figure 4B**). Pearson correlation analysis revealed a strong correlation between *INS* and *GLP1R* copy number in NGFs (p<0.001), and a trend in RHCs (p=0.059) (**Figure 4C**).

## Discussion

Our study shows that a subset of human cortical interneurons—specifically NGFs and RHCs—co-express *GLP1R* and *INS* mRNA, indicating cell-type-specific co-localisation of key metabolic signalling components within inhibitory cortical circuits. This observation is of interest in light of accumulating evidence that metabolic signalling pathways, including insulin signalling and GLP-1 receptor activation, contribute to cognitive function and neurodegenerative processes (8, 9). Experimental studies have demonstrated neuroprotective effect of GLP-1 receptor agonists and improvements in learning and memory in animal models of Alzheimer’s disease (28, 29) and diabetes-associated cognitive impairment (30). Some clinical evidence suggests that they may influence cognitive outcomes in patients with metabolic dysfunction (10, 12). In parallel insulin resistance and disrupted glucose metabolism - features of systemic metabolic disease - have been linked to increased risk of cognitive decline and dementia (32, 33). These observations provide a context in which characterising the local expression of *GLP1R and INS* in human cortical interneurons may be relevant to understanding broader metabolic influences on brain function.

NGFs are relatively uncommon in rodents but eight-times more abundant in the primate cerebral cortex (20). RHCs have so far been identified only in the human neocortex and have not been described in rodents. RHCs display a distinct transcriptomic profile characterised by the expression of **LAMP5, SV2C, and PRSS12**, whereas NGFs exhibit a more conserved molecular signature, robustly expressing **LAMP5** and **SV2C** together with the transcription factor **LHX6**, but lacking **PRSS12** expression (22). Consistent with this, LAMP5-positive interneurons account for approximately 20% of layer 1 inhibitory neurons in the human neocortex, compared with 5–10% in the mouse (23, 24).

Our data indicate that *INS* mRNA is preferentially detected in NGFs and RHCs within the LAMP5-positive population, with *INS* expression essentially absent from other LAMP5-positive interneurons. Although this observation does not establish a functional role, it suggests that these interneuron subtypes may contribute to the *INS* signal detected in human cortical tissue.

This finding is consistent with, and extends, previous reports in rodent systems suggesting local neuronal expression of metabolic-related transcripts (14, 15), and supports the possibility that insulin-related signalling components are present within.

*GLP1R* mRNA was detected in NGFs, RHCs, and other LAMP5-positive interneurons, whereas *INS* mRNA expression was present only in NGFs and RHCs. Although causal links cannot be inferred from the present data, this pattern supports the possibility of an intracortical GLP-1– and insulin-related signalling framework that could be relevant in the context of diabetes-associated cognitive impairment. CNS insulin resistance has been implicated in MCI and AD, and alterations in brain insulin signalling are increasingly recognised as mediators of diabetes-associated cognitive decline (34). Postmortem analyses have shown reduced insulin levels and insulin mRNA in the brains of patients with Alzheimer’s disease (35, 39, 40), while other studies report impairments in insulin receptor signalling, including altered phosphorylation states of insulin receptor substrate proteins (8). If cortical interneurons contribute to local insulin signalling, disruptions in their function could underlie some of these effects. Recent single-nucleus transcriptomic and functional studies indicate that early Alzheimer’s disease pathology is associated with a selective loss of LAMP5-expressing interneurons in the human cortex, contributing to neuronal network dysfunction (36, 37). This highlights the vulnerability of this cell population and underscores the potential functional consequences of impairments in interneuron-derived insulin signalling during disease progression.

We observed high mutual information between *GLP1R* and *INS* mRNA expression indicating a non-random pattern of co-expression at the single-cell level. While the underlying mechanism cannot be inferred from the present data, this association is compatible with coordinated transcriptional regulation or shared cellular state.

We did not observe significant differences in gene expression based on intraoperative blood glucose levels or the presence of brain tumours. This suggests that the observed expression patterns are robust across varied clinical backgrounds, though larger studies are needed to assess potential modulating factors such as chronic hyperglycaemia or neuroinflammation.

Clinical and preclinical studies suggest that GLP-1 receptor agonists may exert beneficial effects on cognitive function in individuals with and without type 2 diabetes; however, clinical evidence remains heterogeneous and largely indirect (37, 38). It is therefore unclear whether reported cognitive effects reflect direct central actions or secondary improvements in systemic metabolic status.

Our finding that *INS* and *GLP1R* mRNA co-localise in specific human cortical interneuron subtypes provides a potential cellular substrate for direct intracortical metabolic signalling. Nevertheless, further studies are required to determine whether and how GLP-1 receptor activation interacts with local insulin signalling within these interneurons to influence cortical function.

Our methodology - combining digital PCR and RNAscope with molecular cell typing -provides a high-resolution view of gene expression at the single-cell level. Different transcriptomic technologies vary considerably in their sensitivity. Digital PCR offers the highest sensitivity of all platforms, with the ability to reliably detect transcript levels with a precision of +/- 1 copy per cell (26). This sensitivity surpasses that of conventional single-cell RNA sequencing platforms such as 10x Genomics or Drop-seq, which typically detect genes expressed at levels above 10–100 copies per cell (40). Consequently, our platform is well-suited for characterising low-abundance transcripts such as *INS* in rare neuronal subtypes. While these methods robustly detect mRNA, future studies should incorporate protein-level validation and functional assays to confirm the roles of neuron-derived insulin in synaptic or metabolic regulation. The question of how many transcript copies are required for functionally relevant protein expression is context-dependent, but studies suggest that 5–10 mRNA copies can suffice for many regulatory or receptor proteins. For instance, Csajbók et al. (15) demonstrated that <10 copies of GLP1R mRNA in cortical interneurons were sufficient for electrophysiologically detectable functional response in single cells. Given that our average *INS* copy number in GNFCs (5.58 ± 2.14) falls within this range, it is likely sufficient to support functional insulin protein synthesis in this neuronal subtype. These findings underscore the biological significance of even low-abundance transcripts when assessed with highly sensitive platforms especially when considering human specific enrichment of neurogliaform-like cells in the cerebral cortex as discussed above.

We report subtype-specific expression and co-localisation of *GLP1R* and *INS* mRNA in human cortical interneurons, particularly in NGFs and RHCs. These findings suggest that the human cortex harbours a local GLP1R-insulin system within its inhibitory circuitry. Further studies are needed to explore the physiological and pathological significance of this system, especially in the context of diabetes mellitus and cognitive health.

## Data Availability

The original contributions presented in the study are included in the article, further inquiries can be directed to the corresponding author.

## References

[1] Kullmann S Heni M Hallschmid M Fritsche A Preissl H Häring HU . Brain insulin resistance at the crossroads of metabolic and cognitive disorders in humans. Physiol Rev. (2016) 96:1169–209. doi: 10.1152/physrev.00032.2015, PMID: 27489306

[2] Kleinridders A Ferris HA Cai W Kahn CR . Insulin action in brain regulates systemic metabolism and brain function. Diabetes. (2014) 63:2232–43. doi: 10.2337/db14-0568, PMID: 24931034 PMC4066341

[3] Schmidt WE Siegel EG Creutzfeldt W . Glucagon-like peptide-1 but not glucagon-like peptide-2 stimulates insulin release from isolated rat pancreatic islets. Diabetologia. (1985) 28:704–7. doi: 10.1007/BF00291980, PMID: 3905480

[4] Mojsov S Weir GC Habener JF . Insulinotropin: glucagon-like peptide I (7-37) co-encoded in the glucagon gene is a potent stimulator of insulin release in the perfused rat pancreas. J Clin Invest. (1987) 79:616–9. doi: 10.1172/JCI112855, PMID: 3543057 PMC424143

[5] Drucker DJ Philippe J Mojsov S WL C Habener JF . Glucagon-like peptide I stimulates insulin gene expression and increases cyclic AMP levels in a rat islet cell line. Proc Natl Acad Sci U S A. (1987) 84:3434–8. doi: 10.1073/pnas.84.10.3434, PMID: 3033647 PMC304885

[6] Göke R Conlon JM . Receptors for glucagon-like peptide-1(7-36) amide on rat insulinoma-derived cells. J Endocrinol. (1988) 116:357–62. doi: 10.1677/joe.0.1160357, PMID: 2832504

[7] Thorens B . Expression cloning of the pancreatic beta cell receptor for the gluco-incretin hormone glucagon-like peptide 1. PNAS. (1992) 89:8641–5. doi: 10.1073/pnas.89.18.8641, PMID: 1326760 PMC49976

[8] Talbot K Wang HY Kazi H Han LY Bakshi KP Stucky A . Demonstrated brain insulin resistance in Alzheimer’s disease patients is associated with IRS-1 dysregulation, cognitive decline, and neurodegeneration. J Clin Invest. (2012) 122:1316–38. doi: 10.1172/JCI59903, PMID: 22476197 PMC3314463

[9] De Felice FG . Alzheimer’s disease and insulin resistance: translating basic science into clinical applications. J Clin Invest. (2013) 123:531–9. doi: 10.1172/JCI64595, PMID: 23485579 PMC3561831

[10] De Giorgi R McCullough LD Rogers JT . Glucagon-like peptide-1 receptor agonists and neurodegenerative disorders: mechanistic and clinical perspectives. J Neurol Neurosurg Psychiatry. (2025) 96:870–83. doi: 10.1136/jnnp-2024-335593, PMID: 40210453 PMC12418562

[11] Craft S Raman R Chow TW Rafii MS Sun C-K Rissman RA . Safety, efficacy, and feasibility of intranasal insulin for cognitive impairment. JAMA Neurol. (2020) 77:1099–109. doi: 10.1001/jamaneurol.2020.1840, PMID: 32568367 PMC7309571

[12] Edison P . Liraglutide in mild to moderate Alzheimer’s disease: a phase 2b clinical trial. Nat Med. (2026) 32:353–61. doi: 10.1038/s41591-025-04106-7, PMID: 41326666 PMC12823385

[13] Cummings JL Lee G Zhong K Fonseca J Taghva K . Alzheimer’s disease drug development pipeline: EVOKE and EVOKE+ trials of semaglutide. Alzheimers Dement. (2024) 20:e12456. doi: 10.1002/alz.12456, PMID: 34515411 PMC9292954

[14] Molnár G Faragó N Kocsis ÁK Rózsa M Lovas S Boldog E . GABAergic neurogliaform cells represent local sources of insulin in the cerebral cortex. J Neurosci. (2014) 34:1133–7. doi: 10.1523/JNEUROSCI.4082-13.2014, PMID: 24453306 PMC6705313

[15] Csajbók ÉA Kocsis ÁK Faragó N Furdan S Kovács B Lovas S . Expression of GLP-1 receptors in insulin-containing interneurons of rat cerebral cortex. Diabetologia. (2019) 62:717–25. doi: 10.1007/s00125-018-4803-z, PMID: 30637442

[16] Tremblay R Lee S Rudy B . GABAergic interneurons in the neocortex: from cellular properties to circuits. Neuron. (2016) 91:260–92. doi: 10.1016/j.neuron.2016.06.033, PMID: 27477017 PMC4980915

[17] Loomba S Straehle J Gangadharan V Heike N Khalifa A Motta A . Connectomic comparison of mouse and human cortex. Science. (2022) 377:eabo0924. doi: 10.1126/science, PMID: 35737810

[18] Fang R Xia C Close JL Zhang M He J Huang Z . Conservation and divergence of cortical cell organization in human and mouse revealed by MERFISH. Science. (2022) 377:56–62. doi: 10.1126/science, PMID: 35771910 PMC9262715

[19] Jorstad NL Song JHT Exposito-Alonso D Suresh H Castro-Pacheco N Krienen FM . Comparative transcriptomics reveals human-specific cortical features. Science. (2023) 382:eade9516. doi: 10.1126/science.ade9516, PMID: 37824638 PMC10659116

[20] Krienen FM Goldman M Zhang Q Del Rosario RCH Florio M Machold R . Innovations present in the primate interneuron repertoire. Nature. (2020) 588:E17. doi: 10.1038/s41586-020-2874-8, PMID: 33230336

[21] Meyer K Kaplan JT . Cross-modal multivariate pattern analysis. J Vis Exp. (2011) 9:3307. doi: 10.3791/3307, PMID: 22105246 PMC3308596

[22] Boldog E Bakken TE Hodge RD Novotny M Aevermann BD Baka J . Transcriptomic and morphophysiological evidence for a specialized human cortical GABAergic cell type. Nat Neurosci. (2018) 21:1185–95. doi: 10.1038/s41593-018-0205-2, PMID: 30150662 PMC6130849

[23] Hodge RD Bakken TE Miller JA Smith KA Barkan ER Graybuck LT . Conserved cell types with divergent features in human versus mouse cortex. Nature. (2019) 573:61–8. doi: 10.1038/s41586-019-1506-7, PMID: 31435019 PMC6919571

[24] Yao Z van Velthoven CTJ Nguyen TN Goldy J Sedeno-Cortes AE Baftizadeh F . A taxonomy of transcriptomic cell types across the isocortex and hippocampal formation. Cell. (2021) 184:3222–3241.e26. doi: 10.1016/j.cell.2021.04.021, PMID: 34004146 PMC8195859

[25] Gouwens NW Sorensen SA Berg J Lee C Jarsky T Ting J . Classification of electrophysiological and morphological neuron types in the mouse visual cortex. Nat Neurosci. (2019) 22:1182–95. doi: 10.1038/s41593-019-0417-0, PMID: 31209381 PMC8078853

[26] Faragó N Kocsis ÁK Lovas S Molnár G Boldog E Rózsa M . Digital PCR to determine the number of transcripts from single neurons after patch-clamp recording. Biotechniques. (2013) 54:327–36. doi: 10.2144/000114029, PMID: 23750542

[27] Brasko C Smith K Molnar C Farago N Hegedus L Balind A . Intelligent image-based in *situ* single-cell isolation. Nat Commun. (2018) 9:226. doi: 10.1038/s41467-017-02628-4, PMID: 29335532 PMC5768687

[28] Perry T Haughey NJ Mattson MP Egan JM Greig NH . Protection and reversal of excitotoxic neuronal damage by glucagon-like peptide-1 and exendin-4. J Pharmacol Exp Ther. (2002) 302:881–8. doi: 10.1124/jpet.102.037481, PMID: 12183643

[29] McClean PL Hölscher C . Liraglutide can reverse memory impairment, synaptic loss and reduce plaque load in aged APP/PS1 mice, a model of Alzheimer’s disease. Neuropharmacology. (2014) 76 Pt A:57–67. doi: 10.1016/j.neuropharm.2013.08.005, PMID: 23973293

[30] Peng X Shi X Huang J Zhang S Yan Y Ma D . Exendin-4 improves cognitive function of diabetic mice via increasing brain insulin synthesis. Curr Alzheimer Res. (2021) 18:546–57. doi: 10.2174/1567205018666210929150004, PMID: 34587885

[31] Zhou T Tang H Zhang B Zhang D Lu Y Li L . Association between glucagon-like peptide-1 receptor agonists and risk of dementia in older adults with type 2 diabetes: A target trial emulation. Diabetes Obes Metab. (2026) 28:1984–96. doi: 10.1111/dom.70384, PMID: 41424236 PMC12890729

[32] Arnold SE Arvanitakis Z Macauley-Rambach SL Koenig AM Wang HY Ahima RS . Brain insulin resistance in type 2 diabetes and Alzheimer disease: concepts and conundrums. Nat Rev Neurol. (2018) 14:168–81. doi: 10.1038/nrneurol.2017.185, PMID: 29377010 PMC6098968

[33] Biessels GJ Despa F . Cognitive decline and dementia in diabetes mellitus. Lancet Neurol. (2018) 17:407–19. 10.1038/s41574-018-0048-7 PMC6397437 30022099

[34] Steen E Terry BM Rivera EJ Cannon JL Neely TR Tavares R . Impaired insulin and insulin-like growth factor expression and signaling mechanisms in Alzheimer’s disease--is this type 3 diabetes? J Alzheimers Dis. (2005) 7:63–80. doi: 10.3233/jad-2005-7107, PMID: 15750215

[35] Gazestani V Kamath T Nadaf NM Dougalis A Burris SJ Rooney B . Early Alzheimer’s disease pathology in human cortex involves transient cell states. Cell. (2023) 186:4438–4453.e23. doi: 10.1016/j.cell.2023.08.005, PMID: 37774681 PMC11107481

[36] Deng Y Bartosovic M Kukanja P Zhang D Liu Y Su G . Spatial-CUT&Tag: Spatially resolved chromatin modification profiling at the cellular level. Science. (2022) 375:681–6. doi: 10.1126/science.abg7216, PMID: 35143307 PMC7612972

[37] Monney M Jornayvaz FR Gariani K . GLP-1 receptor agonists effect on cognitive function in patients with and without type 2 diabetes. Diabetes Metab. (2023) 49:101470. doi: 10.1016/j.diabet.2023.101470, PMID: 37657738

[38] Biswas R Capuano AW Mehta RI Barnes LL Bennett D Arvanitakis Z . Review of associations of diabetes and insulin resistance with brain health in three harmonised cohort studies of ageing and dementia. Diabetes Metab Res Rev. (2025) 41:e70032. doi: 10.1002/dmrr.70032, PMID: 39873127 PMC11774135

[39] Frölich L Blum-Degen D Bernstein HG Engelsberger S Humrich J Laufer S . Brain insulin and insulin receptors in aging and sporadic Alzheimer’s disease. J Neural Transm. (1998) 105:423–38. doi: 10.1007/s007020050068, PMID: 9720972

[40] Macosko EZ Basu A Satija R Nemesh J Shekhar K Goldman M . Highly parallel genome-wide expression profiling of individual cells using nanoliter droplets. Cell. (2015) 161:1202–14. doi: 10.1016/j.cell.2015.05.002, PMID: 26000488 PMC4481139

