# Supplementary material for: Local co-expression of GLP1R and INS in human cortical interneurons
Source: Front Endocrinol (Lausanne). 2026 Mar 27;17:1788432. doi: 10.3389/fendo.2026.1788432 (PMC13065713; doi:10.3389/fendo.2026.1788432)
Supplement: Supplementary file 1 [file Table1.docx]

# Supplementary Table 1. Clinical characteristics of patients

| Case | Age | Sex | Cortical area | Diagnosis | Diabetes mellitus | Intraoperative glucose (mmol/L) | Blood glucose level (mmol/L) | Seizures | Anticonvulsant medication |
| --- | --- | --- | --- | --- | --- | --- | --- | --- | --- |
| 1 | 57 | F | Right temporal gyrus | Tumor | Yes | 9.4 | — | No | None |
| 2 | 69 | M | Right occipital gyrus | Hydrocephalus | No | 5.7 | — | No | None |
| 3 | 56 | M | Right supraciliaris gyrus | Aneurysm | — | 9.4 | 6.4 | No | None |
| 4 | 41 | M | Right temporal gyrus | Hydrocephalus | Yes | 9.2 | 7.1 | No | None |
| 5 | 53 | M | Right temporal gyrus | Tumor | No | 4.7 | — | No | None |
| 6 | 58 | F | Left temporal gyrus | Tumor | No | — | 4.3 | Yes | Levetiracetam, dexamethasone |
| 7 | 68 | M | Right parietal gyrus | Hydrocephalus | Yes | 13 | — | No | — |
| 8 | 66 | F | Right temporal gyrus | Hydrocephalus | Yes | 11.2 | — | No | — |
